# Circ_0000144 functions as a miR-623 sponge to enhance gastric cancer progression via up-regulating GPRC5A

**DOI:** 10.1042/BSR20201313

**Published:** 2020-08-13

**Authors:** Lili Mi, Lianhui Lei, Xiaolei Yin, Ning Li, Jianfei Shi, Xin Han, Xiaoling Duan, Man Zhao, Guangjie Han, Jinfeng Wang, Fei Yin

**Affiliations:** 1 Department of Gastroenterology, the Fourth Hospital of Hebei Medical University, Shijiazhuang, Hebei 050011, China; 2 Department of Surgery, China Electronics Technology Group Corporation No.54 Staff Hospital, Shijiazhuang, Hebei 050011, China

**Keywords:** circ_0000144, GC, GPRC5A, miR-623

## Abstract

**Background:** Gastric cancer (GC) remains one of the most common malignancies worldwide. Increasing evidence has demonstrated that circRNAs serve as critical roles in human cancer, including GC. In the present study, we focused on the detailed function and mechanism of circ_0000144 on GC progression.

**Methods:** The levels of circ_0000144, miR-623 and G-protein-coupled receptor, family C, group 5, member A (GPRC5A) were determined by quantitative real-time polymerase chain reaction (qRT-PCR). Targeted relationships among circ_0000144, miR-623 and GPRC5A were confirmed using dual-luciferase reporter and RNA immunoprecipitation (RIP) assays. Cell proliferation, colony formation, apoptosis, migration and invasion were evaluated by the 3-(4,5-dimethylthiazol-2-yl)-2,5-diphenyltetrazolium bromide (MTT), colony formation, flow cytometry and transwell assays. Measurement of glutamine and α-ketoglutarate (α-KG) levels was performed using a corresponding assay kit. GPRC5A protein expression was detected using Western blot. *In vivo* assays were used to explore the impact of circ_0000144 on tumor growth.

**Results:** Our data indicated that circ_0000144 was up-regulated and miR-623 was down-regulated in GC tissues and cells. Circ_0000144 interacted with miR-623 through directly binding to miR-623. Moreover, the knockdown of circ_0000144 weakened GC cell proliferation, colony formation, migration, invasion and glutaminolysis and accelerated cell apoptosis by up-regulating miR-623. GPRC5A was a direct target of miR-623 and circ_0000144 protected against GPRC5A repression through sponging miR-623. Furthermore, miR-623-mediated regulation on GC cell progression was reversed by the stored expression of GPRC5A. Additionally, circ_0000144 depletion inhibited tumor growth *in vivo.*

**Conclusion:** Our study indicated that circ-0000144 knockdown repressed GC progression at least partly by regulating GPRC5A expression via sponging miR-623, illumining a novel therapeutic target for GC treatment.

## Introduction

Gastric cancer (GC) is the fifth most common cancer and the third leading cause of cancer-related death worldwide in 2018 [1]. The incidence presents a wide geographical variation, and there is a high-risk in China [2,3]. Despite the improved understanding of pathological mechanisms and the advances of therapeutic methods, GC remains a malignant tumor with a generally poor long-term prognosis [2]. This highlights the importance of identifying novel diagnostic and therapeutic targets for GC management.

Glutaminolysis, which catabolizes glutamine to generate ATP and lactate, is a metabolic pathway that plays a critical role in cancer biology. Glutaminolysis accelerates cancer progression through enhancing cell proliferation and repressing cell death [4,5]. Accumulating researches have demonstrated that glutaminolysis is involved in GC progression [6,7]. However, the underlying mechanisms remain largely unknown.

Circular RNAs (circRNAs), an emerging new type of non-coding transcripts, are characterized by a covalently closed loop structure without 5′ caps and 3′ tails [8]. Increasing evidence has demonstrated that circRNAs modulate gene expression through functioning as efficient microRNA (miRNA) sponges, and thus serve as critical roles in human cancer [9,10]. Previous studies had uncovered that a number of circRNAs were associated with GC progression [11]. For example, Zhang et al*.* reported that hsa_circ_0004771 contributed to GC development by sponging miR-149-5p and protecting against AKT serine/threonine kinase 1 (AKT1) repression [12]. Liu and colleagues highlighted that circRNA zinc finger RNA (circ-ZFR) acted as sponges of miR-130a and miR-107 to suppress GC progression via regulating phosphatase and tensin homolog (PTEN) expression [13]. As for circ_0000144, derived from the back-splicing of signaling lymphocyte activation molecules family member 6 (SLAMF6) first intron, it was discovered to act as a tumor promoter in GC and bladder cancer [14,15]. In the present study, we focused on the detailed function of circ_0000144 in GC progression and the mechanism governing it.

MiR-623 has been demonstrated to play a tumor-suppressive role in a series of human cancers, such as pancreatic cancer and lung adenocarcinoma [16,17]. Recent research uncovered that miR-623 was down-regulated in GC, and the elevated expression of miR-623 weakened GC cell growth and promoted chemotherapeutic sensitivity by targeting cyclin D1 [18]. When we used the CircInteractome database to predict the putative miRNAs that bind to circ_0000144, we found a target sequence for miR-623 in circ_0000144. However, whether miR-623 acted as a molecular mediator of circ_0000144 in GC is still undefined.

In the present study, our data supported that circ_0000144 was up-regulated and miR-623 was down-regulated in GC. Consequently, we further investigated the influence and mechanism of circ_0000144 on GC cell proliferation, colony formation, migration, invasion, apoptosis and glutaminolysis.

## Materials and methods

### Tissue specimens and cells

In the present study, 50 GC patients who were pathologically confirmed and had no previous history of other cancers were enrolled from the Fourth Hospital of Hebei Medical University. GC tissues and matched noncancerous tissues were collected from these patients and stored at −80°C until RNA extraction. The tumor-node-metastasis (TNM) classification of these tissues was performed using Union for International Cancer Control (UICC) and American Joint Committee on Cancer (AJCC), and cancer tissues were classified into well-differentiated, moderate-differentiated and undifferentiated according to the degree of glandular differentiation. Participating volunteers signed informed consent, and the use of tissue specimens was approved by the Ethics Committee of the Fourth Hospital of Hebei Medical University.

Human gastric epithelium GES-1 cells, and GC cells HGC-27 and AGS (all from Bnbio, Beijing, China) were grown at 37°C/5% CO_2_ in RPMI-1640 medium (Gibco, Irvine, U.K.) plus 10% fetal bovine serum (FBS, Biosera, Boussens, France) and 1% streptomycin/penicillin (Gibco).

### Quantitative real-time polymerase chain reaction (qRT-PCR)

The miRNeasy Mini Kit (Qiagen, Crawley, U.K.) was used to extract total RNA from GC tissues and cells as recommended by the manufacturers. Afterward, RNA extracts (1 μg) was reverse-transcribed into cDNA using the Qiagen Omniscript RT kit for circ_0000144 and GPRC5A and miScript RT Kit for miR-623. cDNA was then subjected to qRT-PCR using the Qiagen SYBR Green PCR Kit based on the producer’s suggestion. The internal control for normalization was β-actin or U6. The following primers were used: circ_0000144 sense: 5′-GAGCAAATTTGGAGCAAAGG-3′ and antisense: 5′-GGGCCTAAGCTAGTCCCTCA-3′, GPRC5A sense: 5′-AGACAGGGGACACGCTCTAT-3′ and antisense: 5′-GGAGGCAAACTGTTCCCGTA-3′, β-actin sense: 5′-CTCGCCTTTGCCGATCC-3′ and antisense: 5′-GGGGTACTTCAGGGTGAGGA-3′, miR-623 sense: 5′-GCCGAGTGGGTTGTCGGGGACG-3′ and antisense: 5′-CAGTGCGTGTCGTGGAGT-3′, U6 sense: 5′-CTCGCTTCGGCAGCACATATACT-3′ and antisense: 5′-ACGCTTCACGAATTTGCGTGTC-3′. Relative levels of circ_0000144, miR-623 and GPRC5A were evaluated by the standard 2^−ΔΔCt^ method [19].

### Oligonucleotide, plasmid and lentiviral vector transfection

Lentiviral vectors encoding shRNA targeting circ_0000144 (sh-circ_0000144, 5′-AGAAUCUGCUUAGUUCUACCU-3′) or nontarget shRNA (sh-NC, 5′-UUCUCCGAACGUGUCACGUTT-3′) were obtained from HanBio (Shanghai, China). The knockdown of circ_0000144 in AGS cells was performed using the transduction of sh-circ_0000144 in the media containing 8 µg/ml polybrene. For the *in vitro* knockdown of circ_0000144, HGC-27 and AGS cells at 50–60% confluence were transiently transfected with siRNA targeting circ_0000144 (si-circ_0000144, 30 nM, 5′-AAAGAGUAACUUCCAUUUGUC-3′, Ribobio, Guangzhou, China) or a control siRNA (si-NC, 5′-AACAGTCGCGTTTGCGACTGG-3′, Ribobio). For GPRC5A overexpression *in vitro*, the sequence of GPRC5A containing the 3′-untranslated region (3′-UTR) was cloned into a pcDNA3.1 vector to generate pcDNA-based GPRC5A overexpression plasmid (pc-GPRC5A, Ribobio) and then 20 ng of the plasmid was transfected into cells, with a nontarget pcDNA plasmid (pc-NC, Ribobio) as the negative control. The alteration of miR-623 expression was carried out using synthetic miR-623 mimic (30 nM, 5′-UGGGUUGUCGGGGACGUUCCCUA-3′, Ribobio), miR-623 inhibitor (30 nM, 5′-UAGGGAACGUCCCCGACAACCCA-3′, Ribobio), or a corresponding scrambled sequence (miRNA NC mimic, 5′-UUCUCCGAACGUGUCACGUTT-3′ or inhibitor NC, 5′-CAGUACUUUUGUGUAGUACAA-3′, Ribobio). Each transfection was conducted using the commercial Lipofectamine RNAiMAX reagent (Invitrogen, Burlington, ON, Canada) as recommended by the manufacturers.

### Bioinformatics

The online CircInteractome database (https://circinteractome.nia.nih.gov) was used for the prediction of the miRNAs that putatively pair to circ_0000144. Analysis for miR-623 targets was implemented using the TargetScan software (http://www.targetscan.org/vert_71/).

### Dual-luciferase reporter and RNA immunoprecipitation (RIP) assays

The segmental sequence of circ_0000144 harboring the miR-623-pairing sites, the 3′-untranslated region (3′-UTR) of GPRC5A and their mutants in the seed sites were inserted by restriction endonuclease into the pmirGLO vector (Promega, Charbonnières, France), respectively, as described previously [20]. These constructs were transfected into HGC-27 and AGS cells, respectively, together with miR-623 mimic or miRNA NC mimic and tested for luciferase activity using the Promega Dual-luciferase Reporter Assay Kit. *Renilla* and Firefly luciferase activities were tested 48 h post-transfection using a luminometer (Berthold, Thoiry, France) based on the guidance of producers.

In RIP assays, the preparation of cell lysates was performed using the Complete Lysis-M reagent (Roche, Sussex, U.K.). After that, cell lysates were incubated with antibody against Argonaute2 (anti-Ago2, ab156870, Abcam, Cambridge, U.K.) or IgG (anti-IgG, ab150077, Abcam) for 2 h at 4°C before adding the protein A/G agarose (Sigma-Aldrich, Sydney, NSW, Australia) for 2 h. Beads were harvested, and total RNA was isolated, followed by the detection of the levels of circ_0000144, miR-623 and GPRC5A.

### Cell proliferation, colony formation and apoptosis assays

HGC-27 and AGS cells were carried out the indicated transfections as described above. After 24, 48 and 72 h transfection, the 3-(4,5-Dimethylthiazol-2-yl)-2,5-diphenyltetrazolium bromide (MTT) colorimetric assay was used for the determination of cell proliferation as reported previously [21]. The amount of formazan produced was proportional to the number of viable cells, which was tested by a spectrophotometer (Thermo Fisher Scientific, Wesel, Germany) at 490 nm. In colony formation assay, transfected cells were grown in 6-well plates for 15 days, stained with 1% crystal violet (Solarbio, Beijing, China) and then the number of colonies (at least 50 cells) was counted. Apoptosis of the cells after various transfection for 48 h was determined by flow cytometry using Annexin V-FITC/propidium Iodide (PI) staining assay (Beyotime, Shanghai, China) based on the producer’s protocols.

### Cell migration and invasion assays

The transwell migration and invasion assays were performed using the 24-well transwell insert (8 μm) and Matrigel-precoated transwell insert (all from Corning, Rochester, NY, U.S.A.), respectively. About 100 μl of cell solution (containing 5 × 10^5^ cells) was plated into the top chamber of the transwell insert, and 600 μl of 10% FBS medium was added into the lower chamber. Twenty-four hours later, the migrated or invaded cells were fixed with 70% ethanol, stained with 1% Crystal Violet and the number of the cells was counted under a light microscope (100× magnification, Nikon, Tokyo, Japan) in random five fields.

### Measurement of glutamine and α-ketoglutarate (α-KG) levels

Analysis of glutamine level in the cells after 48 h various transfections was done using a Quantitative Colorimetric Enzyme Assay Kit (BioAssay Systems, Brussels, Belgium) as recommended by the producers. The α-KG level was determined in the cells after 48 h transfection using the α-KG assay kit (Abcam) based on the protocols of manufacturers. The untransfected cells were defined as the control group, and the relative levels of glutamine and α-KG were calculated as percentage of control cells.

### Western blot for GPRC5A

Total protein extraction and Western blot assay were conducted as reported previously [22]. Briefly, total protein (50 μg) was resolved on a 10% SDS polyacrylamide gel and transferred to the nitrocellulose membrane (Corning). Western blot was implemented using primary antibodies against GPRC5A (anti-GPRC5A, ab155557) and glyceraldehyde-3-phosphate dehydrogenase (anti-GAPDH, ab181602, all from Abcam).

### *In vivo* assays

All experiments were approved by the Ethics Committee of the Fourth Hospital of Hebei Medical University, and the animal handling was conducted according to the National Guidelines of Animal Care and Use Committee. For *in vivo* animal studies, male 5-week-old BALB/c mice (Guangdong Medical Laboratory Animal Center, Foshan, China) were used and fed under specific pathogen-free conditions. Approximately 5 × 10^6^ AGS cells stably transduced sh-NC or sh-circ_0000144 were subcutaneously injected into the left flank of nude mice. On 7 days after injection, tumor volume was measured with calipers every week. After 28 days of injection, all mice were killed and tumor tissues were collected.

### Statistical analysis

Statistical analyses were done by a Student’s *t*-test and analysis of variance (ANOVA), followed by the Tukey’s post hoc test. Results were expressed as mean ± standard deviation (SD) and considered statistically significant when *P* was < 0.05.

## Results

### Circ_0000144 was up-regulated and miR-623 was down-regulated in GC tissues and cells

**Figure 1:**
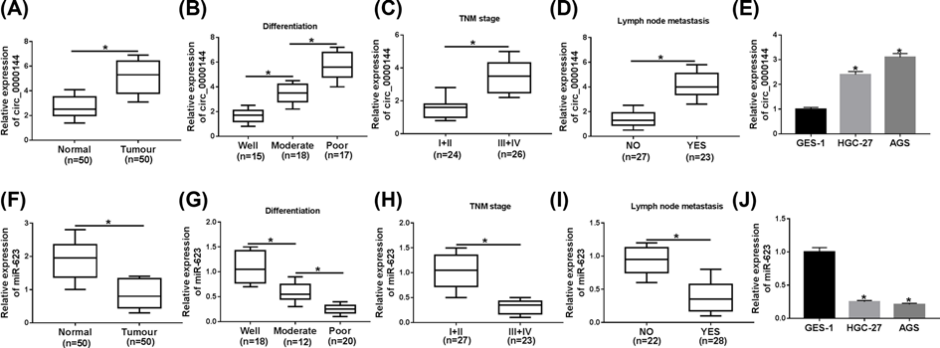
Circ_0000144 was up-regulated and miR-623 was down-regulated in GC tissues and cells (**A**) Circ_0000144 expression was detected by qRT-PCR in 50 pairs of GC tissues and adjacent noncancerous tissues. (**B–D**) The correlations between circ_0000144 and tumor differentiation, TNM stage and lymph node metastasis were analyzed. (**E**) Circ_0000144 expression was assessed by qRT-PCR in GES-1, HGC-27 and AGS cells. (**F**) MiR-623 expression was evaluated by qRT-PCR in GC tissues and adjacent noncancerous tissues. (**G–I**) The correlations between miR-623 and tumor differentiation, TNM stage and lymph node metastasis were determined. (**J**) MiR-623 expression was tested in GES-1, HGC-27 and AGS cells; *P<0.05.

First, we determined the expression of circ_0000144 in GC tissues and cells. As demonstrated by qRT-PCR, circ_0000144 was highly expressed in GC tissues compared with adjacent noncancerous tissues (Figure 1A). Notably, the elevated expression of circ_0000144 was closely correlated with poor cell differentiation (Figure 1B), advanced TNM stage (Figure 1C) and lymph node metastasis (Figure 1D). Moreover, in contrast with the GES-1 cells, circ_0000144 level was elevated in GC cells (Figure 1E). Conversely, miR-623 was significantly down-regulated in GC tissues and cells, and low miR-623 level was correlated with poor cell differentiation, advanced TNM stage and lymph node metastasis (Figure 1F–J).

### Circ_0000144 interacted with miR-623 through directly binding to miR-623

**Figure 2:**
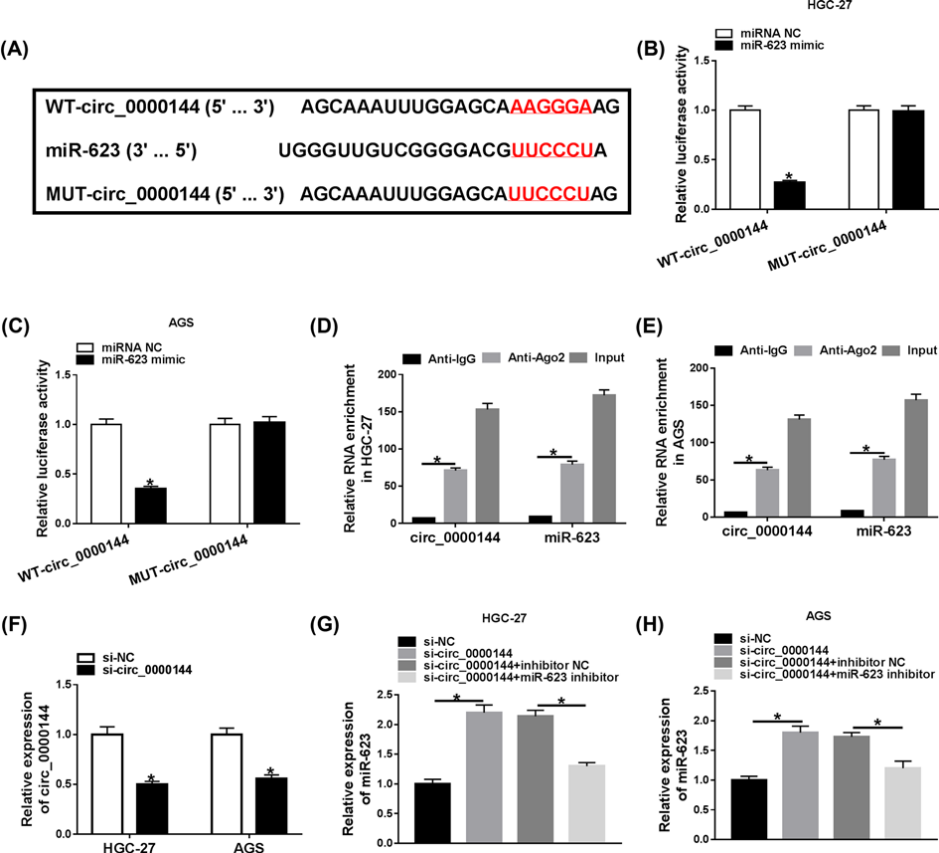
Circ_0000144 interacted with miR-623 through directly binding to miR-623 (**A**) Schematic of the miR-623-binding sites in circ_0000144 and the mutant in the seed sequence. (**B** and **C**) Relative luciferase activity was assessed in the cells cotransfected with WT-circ_0000144 or MUT-circ_0000144 and miR-623 mimic or miRNA NC mimic. (**D** and **E**) The enrichment levels of circ_0000144 and miR-623 were evaluated by qRT-PCR using anti-Ago2 or anti-IgG antibody. (**F**) Circ_0000144 expression was tested in the cells transfected with si-NC or si-circ_0000144. (**G** and **H**) MiR-623 level was detected in the cells transfected with si-NC, si-circ_0000144, si-circ_0000144+inhibitor NC or si-circ_0000144+miR-623 inhibitor; *P<0.05.

Uing the online CircInteractome database, the predicted data revealed that circ_0000144 harbored a putative target sequence for miR-623 (Figure 2A). To validate this, circ_0000144 wild-type reporter construct (WT-circ_0000144) or the mutant in the target region (MUT-circ_0000144) was transfected into the cells together with miR-623 mimic or miRNA NC mimic. Cotransfection of miR-623 mimic, but not the negative sequence, significantly down-regulated the luciferase activity of WT-circ_0000144 (Figure 2B,C). However, the reduced effect of miR-623 on luciferase activity was remarkably abolished upon MUT-circ_0000144 introduction (Figure 2B,C), indicating the validity of the binding sites for interaction. The data of RIP experiments showed that circ_0000144 and miR-623 were simultaneously enriched by anti-Ago2 antibody (Figure 2D,E), implying the endogenous interplay between circ_0000144 and miR-623. Additionally, qRT-PCR results demonstrated that circ_0000144 level was strikingly reduced by the transfection of si-circ_0000144 in both HGC-27 and AGS cells (Figure 2F). Furthermore, miR-623 expression was prominently elevated by circ_0000144 knockdown, and this effect was strongly reversed after the introduction of miR-623 inhibitor (Figure 2G,H). Additionally, circ_0000144 expression was not be affected by miR-623 inhibitor (Supplementary Figure S1).

### Circ_0000144 knockdown suppressed GC cell proliferation, colony formation, migration, invasion and glutaminolysis and enhanced apoptosis by miR-623

**Figure 3:**
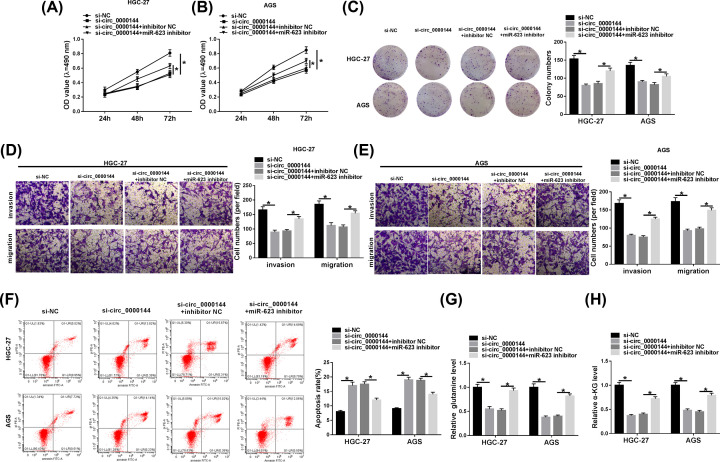
Circ_0000144 depletion repressed GC cell malignant progression via up-regulating miR-623 HGC-27 and AGS cells were transfected with si-NC, si-circ_0000144, si-circ_0000144+inhibitor NC or si-circ_0000144+miR-623 inhibitor, followed by the measurement of cell proliferation by MTT assay (**A** and **B**), cell colony formation using a standard colony formation assay (**C**), cell migration and invasion by transwell assay (**D** and **E**), cell apoptosis by flow cytometry (**F**), the levels of glutamine and α-KG using a corresponding assay kit (**G** and **H**); *P<0.05.

To determine the impact of circ_0000144 on GC cell progression, loss-of-function experiments were carried out by using si-circ_0000144. In comparison with the negative control, MTT and colony formation assays revealed that circ_0000144 depletion resulted in a significant inhibition in cell proliferation (Figure 3A,B) and colony formation (Figure 3C). Transwell analyses showed that circ_0000144 knockdown led to a clear reduction in cell migration and invasion (Figure 3D,E). Flow cytometry demonstrated that cell apoptosis was highly promoted by the silencing of circ_0000144 (Figure 3F). Additionally, circ_0000144 depletion resulted in decreased levels of glutamine (Figure 3G) and α-KG (Figure 3H) in the two cells, indicating its inhibitory effect on cell glutaminolysis. Nevertheless, these effects of circ_0000144 knockdown were remarkably reversed by the transfection of miR-623 inhibitor (Figure 3A–H).

### GPRC5A was directly targeted and inhibited by miR-623

**Figure 4:**
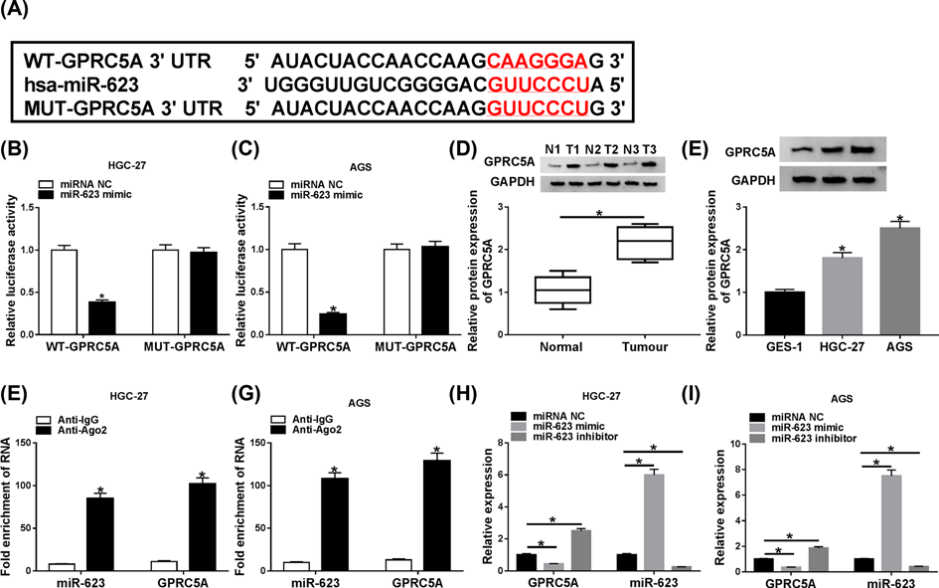
GPRC5A was a direct target of miR-623 (**A**) Schematic model of the complementary sequence for miR-623 in GPRC5A 3′-UTR. (**B** and **C**) The luciferase activity was determined in the cells cotransfected with WT-GPRC5A or MUT-GPRC5A and miR-623 mimic or miRNA NC mimic. (**D** and **E**) GPRC5A expression was evaluated by Western blot in three pairs of GC tissues and adjacent noncancerous tissues, GES-1, HGC-27 and AGS cells. (**F** and **G**) The enrichment levels of miR-623 and GPRC5A were tested when using anti-Ago2 antibody. (**H** and **I**) The levels of miR-623 and GPRC5A were detected by qRT-PCR in HGC-27 and AGS cells transfected with miRNA NC mimic, miR-623 mimic, inhibitor NC or miR-623 inhibitor; *P<0.05.

Then, we conducted a detailed analysis for the molecular targets of miR-623 using the online software TargetScan. The predicted data demonstrated that GPRC5A contained a potential complementary sequence in its 3′-UTR (Figure 4A). With luciferase reporter plasmid (WT-GPRC5A) and miR-623 overexpression caused a striking reduction in luciferase activity (Figure 4B,C). When the target sequence was mutated (MUT-GPRC5A), little change was found in luciferase in the presence of miR-623 mimic (Figure 4B,C). The data of Western blot also revealed that in contrast with their counterparts, GPRC5A was up-regulated in GC tissues and cells (Figure 4D,E). RIP assays showed that the enrichment levels of miR-623 and GPRC5A were markedly elevated by anti-Ago2 antibody (Figure 4F,G). qRT-PCR results presented that miR-623 expression was significantly increased by miR-623 mimic and decreased when transfecting with miR-623 inhibitor in the two cells (Figure 4H,I). Moreover, GPRC5A level was prominently reduced by miR-623 overexpression, and it was highly elevated after the knockdown of miR-623 (Figure 4H,I).

### GPRC5A mediated the regulatory impact of miR-623 overexpression on GC cell progression

**Figure 5:**
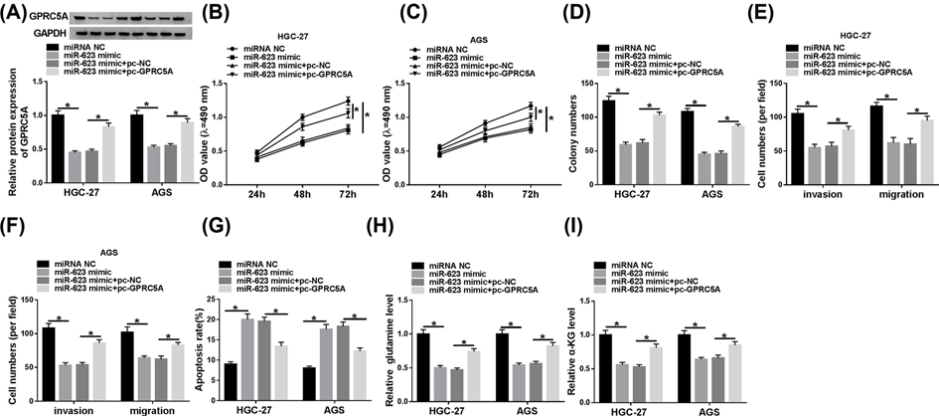
GPRC5A mediated the regulatory impact of miR-623 overexpression on GC cell progression HGC-27 and AGS cells were transfected with miRNA NC mimic, miR-623 mimic, miR-623 mimic+pc-NC or miR-623 mimic+pc-GPRC5A, followed by the detection of GPRC5A expression by Western blot (**A**), cell proliferation by MTT assay (**B** and **C**), cell colony formation using a standard colony formation assay (**D**), cell migration and invasion by transwell assay (**E** and **F**), cell apoptosis by flow cytometry (**G**), the levels of glutamine and α-KG using a corresponding assay kit (**H** and **I**); *P<0.05.

To provide further understand about the link between miR-623 and GPRC5A on GC cell progression, miR-623 mimic and GPRC5A overexpression plasmid (pc-GPRC5A) were cotransfected into the cells. In comparison with the negative control, miR-623 mimic-mediated GPRC5A reduction was significantly abolished by pc-GPRC5A transfection (Figure 5A). Further experiments analyses revealed that miR-623 overexpression led to a striking repression in cell proliferation (Figure 5B,C), colony formation (Figure 5D), migration and invasion (Figure 5E,F), and a distinct enhancement in cell apoptosis (Figure 5G), as well as a strong inhibition in cell glutaminolysis (Figure 5H,I). Moreover, these effects were remarkably reversed by stored GPRC5A expression (Figure 5B–I). Besides, by contrast, the transfection of pc-GPRC5A led to a significant increase in the level of GPRC5A, and the increased GPRC5A expression remarkably enhanced cell proliferation in the two GC cells (Supplementary Figure S2).

### Circ_0000144 modulated GPRC5A through sponging miR-623

**Figure 6:**
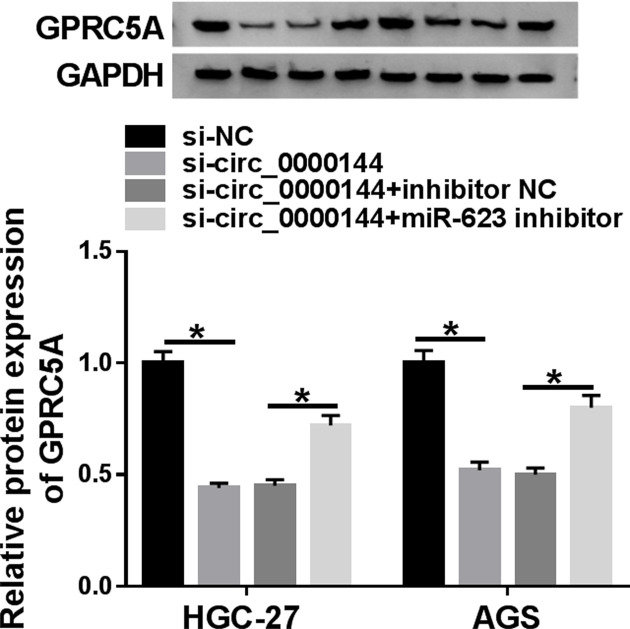
Circ_0000144 modulated GPRC5A through sponging miR-623 GPRC5A expression was assessed by Western blot in the cells transfected with si-NC, si-circ_0000144, si-circ_0000144+inhibitor NC or si-circ_0000144+miR-623 inhibitor *P<0.05.

CircRNAs are widely accepted to mediate gene expression via acting as sponges of miRNAs. Herein, we explored whether circ_0000144 regulated GPRC5A in GC cells. As expected, the data of qRT-PCR revealed that the expression of GPRC5A was markedly decreased when circ_0000144 knockdown, and this effect was prominently reversed by the introduction of miR-623 inhibitor (Figure 6).

### Circ_0000144 knockdown hindered tumor growth *in vivo*

**Figure 7:**
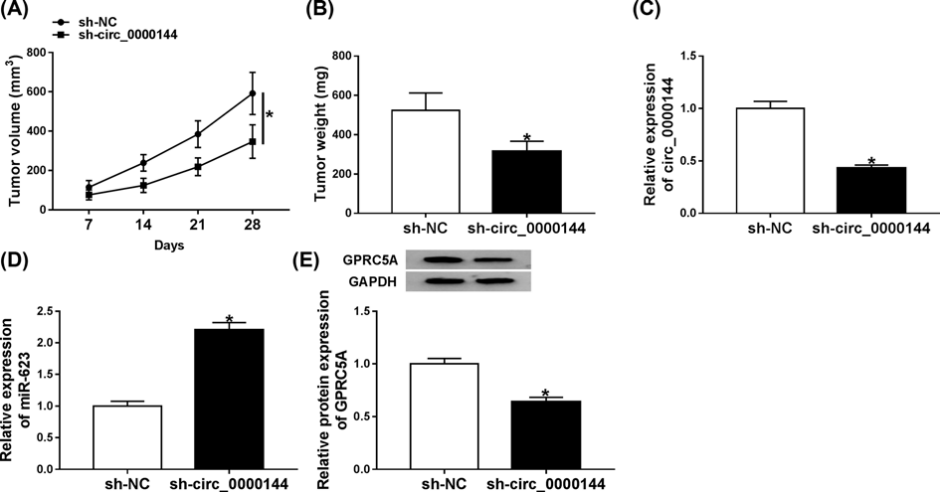
Circ_0000144 depletion hampered tumor growth *in vivo* AGS cells stably transduced sh-NC or sh-circ_0000144 were subcutaneously injected into the nude mice. After 28 days of injection, all mice were killed and tumor tissues were collected. (**A**) On 7 days after injection, tumor volume was measured every week. (**B**) Tumor weight was weighed. The expression levels of circ_0000144 (**C**), miR-623 (**D**), GPRC5A (**E**) were determined by qRT-PCR or Western blot in excised tumor tissues; *P<0.05.

Given our data that circ_0000144 regulated GC cell progression *in vitro*, we further observed its function on tumor growth *in vivo* using the xenograft mice model. These data showed that the transduction of sh-circ_0000144 significantly hindered tumor growth compared with the negative control (Figure 7A,B). Moreover, the data of qRT-PCR and Western blot revealed that circ_0000144 and GPRC5A were dramatically down-regulated and miR-623 was strikingly up-regulated in tumor tissues derived from sh-circ_0000144-transducing cells (Figure 7C–E).

## Discussion

**Figure 8:**
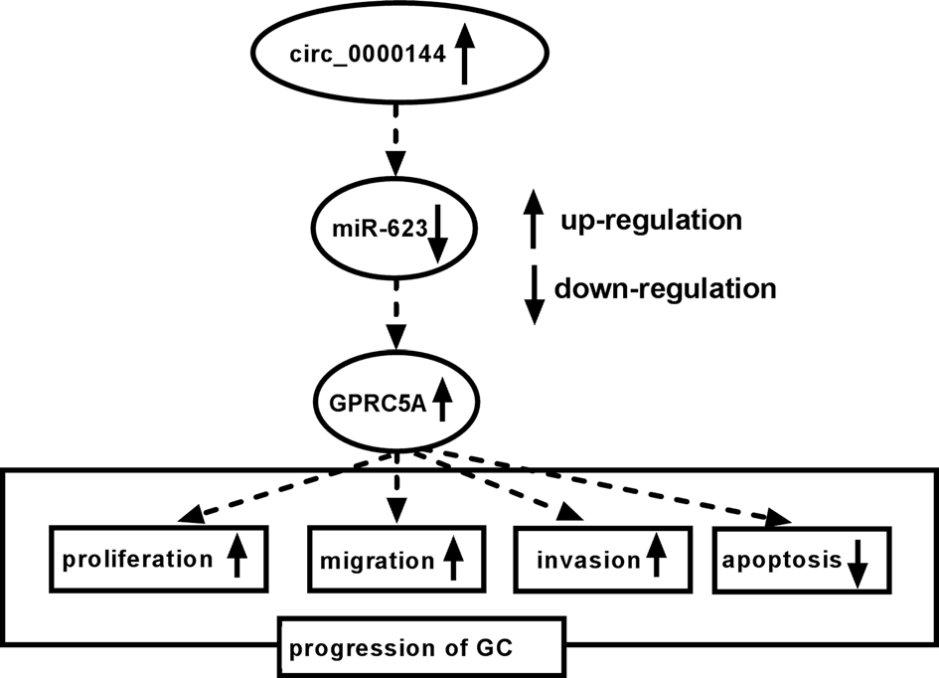
The schematic model of the circ_0000144/miR-623/GPRC5A axis in GC progression In GC tissues and cells, circ_0000144 was up-regulated. Then, the increased circ_0000144 expression reduced the abundance of miR-623, and thus elevated GPRC5A level. Subsequently, the elevated expression of GPRC5A enhanced GC cell proliferation, migration, invasion and inhibited apoptosis, thereby promoting GC progression.

Recently, it has become apparent that a wide variety of circRNAs implicate in tumorigenesis and development of GC, highlighting their potential as diagnostic and prognostic biomarkers and therapeutic targets in future cancer medicine [11]. In the present work, we identified a new circRNA circ_0000144 that was up-regulated in GC. Furthermore, our study had led to the identification of circ_0000144 that modulated GC progression by regulating GPRC5A expression through acting as a miR-623 sponge (Figure 8).

In the current work, we first demonstrated that the elevated expression of circ_0000144 predicted a poor clinical outcome of GC patients. Our work validated that circ_0000144 knockdown mitigated GC cell proliferation, migration and invasion and enhanced apoptosis, consistent with a recent study [14]. Moreover, the depletion of circ_0000144 hindered tumor growth *in vivo*. Glutamine metabolism has been demonstrated to play a crucial role in cancer cell growth and survival, and high glutamine level contributes to tumor development. Glutaminolysis produces α-KG and thus supports cancer cell proliferation and tumor growth [5,23,24]. In the present research, for the first time, we uncovered that circ_0000144 depletion led to a significant inhibition in cell glutaminolysis. In short, circ_0000144 knockdown exerted a tumor-suppressive function in GC. Similar to our findings, Huang et al*.* reported that circ_0000144 silencing hampered bladder cancer cell proliferation and invasion by targeting miR-217/RUNX family transcription factor 2 (RUNX2) axis [15].

It is widely accepted that circRNAs mediate gene expression via acting as sponges of miRNAs during the tumorigenic process [25,26]. Hence, we used the online CircInteractome database to help identify the potentially interacted miRNAs of circ_0000144. Subsequently, we were first to verify that circ_0000144 interacted with miR-623 via directly binding to miR-623. The researches by Chen et al*.* manifested that miR-623 inhibited pancreatic cancer metastasis *in vitro* and *in vivo* through targeting matrix metalloproteinase-1 (MMP1) [16]. Ren and colleagues illuminated that miR-623 functioned as a tumor suppressor in hepatocellular carcinoma through modulating the phosphoinositide 3‐kinase/protein kinase B (PI3K/Akt) pathway via targeting X-ray repair cross complementing 5 (XRCC5) [27]. In the current study, our data demonstrated that low miR-623 level was associated with a poor clinical outcome of GC patients. Moreover, we substantiated that the overexpression of miR-623 repressed GC cell malignant behaviors and accelerated cell apoptosis, in agreement with former work [18]. Furthermore, for the first time, we uncovered that miR-623 mediated the suppressive impact of circ_0000144 knockdown on GC cell progression.

Then, we performed a detailed analysis for miR-623 targets using the TargetScan software. Among these thousands of candidates, GPRC5A was of interest in the current research because its expression had been found to be up-regulated in GC and the elevated expression of GPRC5A predicted advanced clinical features and poor prognosis [28,29]. GPRC5A was also reported to act as a potential oncogene in prostate cancer and pancreatic ductal adenocarcinoma [30,31]. Moreover, GPRC5A was involved in GC cell malignant progression through acting as a direct target of miR-204 and miR-195 [32,33]. In the present research, we were first to confirm that GPRC5A was directly targeted and suppressed by miR-623 in GC cells. Furthermore, miR-623 weakened GC cell malignant progression via GPRC5A. More interestingly, for the first time, we highlighted that circ_0000144 protected against GPRC5A repression through sponging miR-623. Additionally, circ_0000144 knockdown resulted in increased miR-623 expression and decreased GPRC5A level in xenograft tissues *in vivo*. Thus, more *in vivo* investigations about the novel mechanism in GC progression will be carried out in further work. Non-coding RNA (ncRNAs), including circRNAs and miRNAs, have been demonstrated as important regulators of glutaminolysis in cancer biology [34]. In the present study, circ_0000144 regulated GC cell glutaminolysis by targeting miR-623/GPRC5A axis. Future work would build on these findings by determining how the novel mechanism regulated GC cell glutaminolysis.

In conclusion, the present study suggested that the knockdown of circ_0000144 mitigated GC progression via modulating GPRC5A expression by acting as a miR-623 sponge. The findings of the present study have highlighted the potential role of circ_0000144 as a tumor promoter in GC, therefore, serve as a potential therapeutic target for GC treatment.

## Supplementary Material

Supplementary Figures S1-S2

## Abbreviations

AKT1: AKT serine/threonine kinase 1
GC: gastric cancer
MMP1: matrix metalloproteinase-1
PTEN: progression via regulating phosphatase and tensin homolog
RIP: RNA immunoprecipitation
SLAMF6: signaling lymphocyte activation molecules family member 6
