# Correction: Circ_0000144 functions as a miR-623 sponge to enhance gastric cancer progression via up-regulating GPRC5A

**DOI:** 10.1042/BSR-20201313_COR

**Published:** 2020-10-16

**Keywords:** circ_0000144, GC, GPRC5A, miR-623

The authors of the original article “Circ_0000144 functions as a miR-623 sponge to enhance gastric cancer progression via up-regulating GPRC5A” (*Biosci Rep* (2020) 40(8), https://doi.org/10.1042/BSR20201313) would like to correct their Ethics Approval statement. The correct Ethics Approval should be as follows:

The study was approved by the ethics committee of the Fourth Hospital of Hebei Medical University. The patients or their relatives gave written informed consent prior to entering the study.

The authors apologise for any inconvenience or misunderstanding that this error may have caused.

