# Supplementary material for: Circ_0000144 functions as a miR-623 sponge to enhance gastric cancer progression via up-regulating GPRC5A
Source: Biosci Rep. 2020 Aug 13;40(8):BSR20201313. doi: 10.1042/BSR20201313 (PMC7426631; doi:10.1042/BSR20201313)
Supplement: Supplementary Figures S1-S2 [file BSR-2020-1313_supp.pdf]

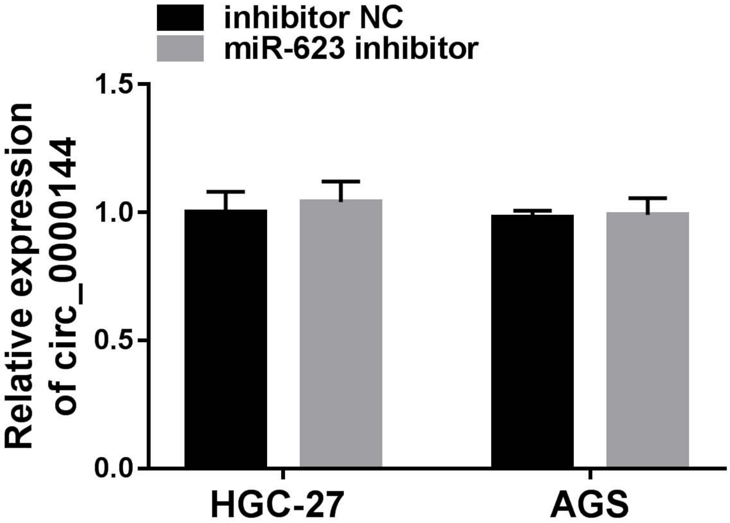

inhibitor NC
miR-623 inhibitor
Relative expression of circ_0000144
1.5
1.0
0.5
0.0
HGC-27
AGS

Supplement Figure 1. The effect of miR-623 on circ_0000144 expression in both AGS and HGC-27 cells.

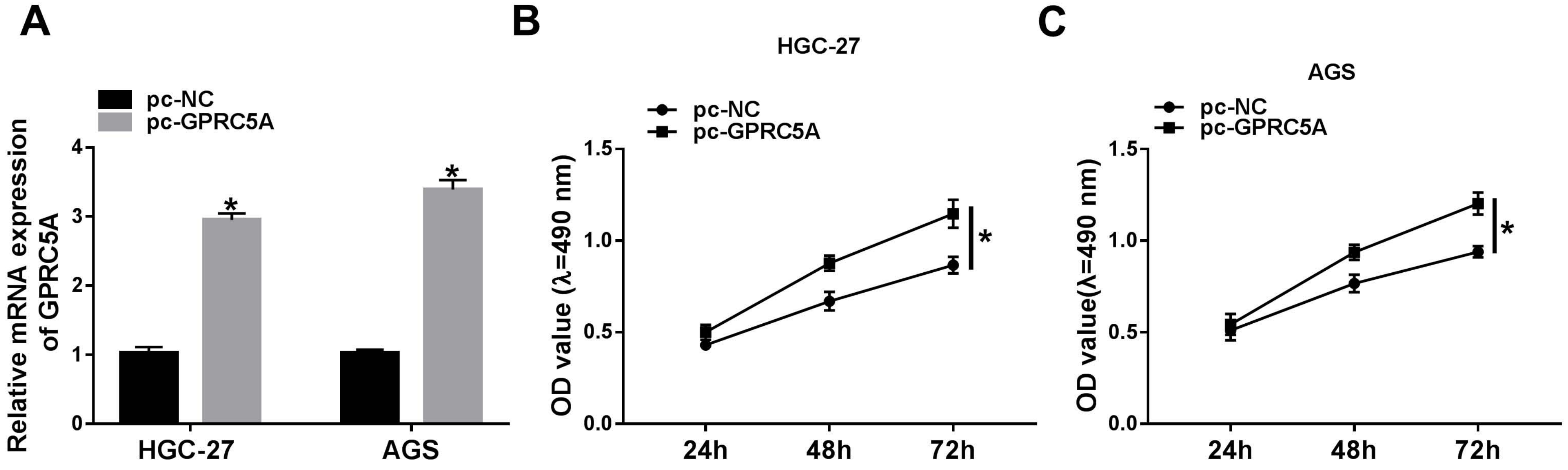
A
pc-NC
pc-GPRC5A
Relative mRNA expression of GPRC5A
4
3
2
1
0
*
*
HGC-27
AGS
B
HGC-27
pc-NC
pc-GPRC5A
OD value (λ=490 nm)
1.5
1.0
0.5
0.0
*
24h
48h
72h
C
AGS
pc-NC
pc-GPRC5A
OD value(λ=490 nm)
1.5
1.0
0.5
0.0
*
24h
48h
72h

Supplement Figure 2. The effect of GPRC5A over expression on cell proliferation. HGC-27 and AGS cells were transfected with pc-GPRC5A or pc-NC, followed by the assessment of GPRC5A expression by qRT-PCR (A), and cell proliferation by MTT assay (B and C). *P< 0.05.
